# Effect of Ca^2+^ Channel Block on Glycerol Metabolism in *Dunaliella salina* under Hypoosmotic and Hyperosmotic Stresses

**DOI:** 10.1371/journal.pone.0028613

**Published:** 2011-12-14

**Authors:** Hui Chen, Shan-Li Chen, Jian-Guo Jiang

**Affiliations:** 1 College of Food and Bioengineering, South China University of Technology, Guangzhou, China; 2 Institute of Hydrobiology, Chinese Academy of Sciences, Wuhan, China; University of Queensland, Australia

## Abstract

The effect of Ca^2+^ channel blockers on cytosolic Ca^2+^ levels and the role of Ca^2+^ in glycerol metabolism of *Dunaliella salina* under hypoosmotic or hyperosmotic stress were investigated using the confocal laser scanning microscope (CLSM). Results showed that intracellular Ca^2+^ concentration increased rapidly when extracellular salinity suddenly decreased or increased, but the increase could be inhibited by pretreatment of Ca^2+^ channel blockers LaCl_3_, verapamil or ruthenium red. The changes of glycerol content and G3pdh activity in *D. salina* to respect to hypoosmotic or hyperosmotic stress were also inhibited in different degrees by pretreatment of Ca^2+^ channel blockers, indicating that the influx of Ca^2+^ via Ca^2+^ channels are required for the transduction of osmotic signal to regulate osmotic responses of *D. salina* to the changes of salinity. Differences of the three blockers in block effect suggested that they may act on different channels or had different action sites, including influx of Ca^2+^ from the extracellular space via Ca^2+^ channels localized in the plasma membrane or from intracellular calcium store via the mitochondrial. Other Ca^2+^-mediated or non-Ca^2+^-mediated osmotic signal pathway may exist in *Dunaliella* in response to hypoosmotic and hyperosmotic stresses.

## Introduction

Many plants are adversely affected by several environmental factors that have a negative effect on their survival and development, such factors are light, temperature, CO_2_, O_2_, water, nutrients, and stresses as drought, low pH, salt, and pathogen or predator attack [1]. Ca^2+^ has been shown to serve as a ubiquitous intracellular second messenger in signal transduction of environmental stimuli in plants [2]–[4]. When plants are forced to respond to environmental stimuli, Ca^2+^ level rises rapidly and transiently in the cytoplasm either as a result of uptake from the extracellular space through plasma membrane channels or of the release from internal stores, such as the endoplasmic reticulum or vacuoles [4]. Free Ca^2+^, and the proteins which bind them, are important and conserved components of intracellular signalling networks [5], [6]. Typical proteins that bind Ca^2+^ are calmodulin (CAM) and Ca^2+^- or CaM-dependent enzymes (e.g., calmodulin-domain protein kinases [CDPKs], calcineurin), which translate Ca^2+^ level changes into the regulation of proteins to produce appropriate response [1], [7]–[11].

*Dunaliella salina* is an extremely halotolerant, unicellular, green, and motile algae, which is unique in its remarkable ability to survive in media containing a wide range of NaCl concentrations, ranging from about 0.05 M to saturation (around 5.5 M), while maintaining a relatively low intracellular sodium concentration [12]. In addition, under high salt stress, *D. salina* could accumulate large amounts of β-carotene in cells, which makes it one of the best sources of natural β-carotene [13]–[16]. The osmotic adjustment response of *Dunaliella* under salt stress functions by varying the intracellular concentration of primarily a single compatible solute, glycerol [17]. The osmotic adaptation is marked by reassumption of the original cell volume due to the accumulation of an osmotically compatible content of glycerol [18]. At high salinity, *Dunaliella* accumulates massive amounts of glycerol and the level of intracellular glycerol was found to be proportional and osmotically equivalent to the external NaCl concentration, reaching about 8 M or 55% of the cell weight at saturated NaCl [19]. G3pdh is an important enzyme in the pathway of glycerol synthesis. In higher plants and algae, G3pdh is referred to as dihydroxyacetone phosphate (DHAP) reductase, because at physiological pH and substrate, the enzyme is essentially inactive as a dehydrogenase [20].

In order to obtain direct evidence of the involvement of Ca^2+^ in the mechanism of osmotic signaling in *Dunaliella*, in the present research the change of cytosolic Ca^2+^ level and the role of Ca^2+^ in glycerol metabolism and G3pdh activity under hypoosmotic or hyperosmotic stress in *D. salina* were investigated using the CLSM and a pharmacological approach. The aim of this paper is to study the role of Ca^2+^ by using Ca^2+^ channel blockers LaCl_3_, verapamil (VP) and ruthenium red (RR) to elucidate the osmotic stress signal transduction pathway in *D. salina*.

## Materials and Methods

### Cultivation of *D. salina*

*D. salina* strain (FACHB-435) was obtained from Freshwater Algae Culture Collection of the Institute of Hydrobiology, Chinese Academic of Sciences. Cells of *D. salina* were cultivated in the culture medium containing 2.0 M NaCl at 26°C and 108 µmol m^−2^ s^−1^ provided by cool-white fluorescent lamps, under a 14/10 h light/dark cycle with shaking at 96 rpm according to Chen et al. [21].

### *D. salina* cell loading with Fluo-3 AM

The algal cells at log phase were harvested by centrifugation at 5,000 g for 15 min at room temperature. The algal pellet was resuspended in fresh iso-osmotic and iso-volumetric medium and cultivated for 1 h. Then *D. salina* cells were loaded with the fluorescent dyes 5 µmol/L final concentration of acetoxymethyl ester form (AM) of fluo-3 and 50 mmol/L final concentration of sorbitol at 25°C in the dark for 1 h. Loaded cells were subsequently rinsed 3 times in fresh iso-osmotic medium, and then harvested by centrifugation. Harvested loaded cells were resuspended in fresh iso-osmotic and iso-volumetric medium and cultivated for 2 h again.

### Pretreatment with Ca^2+^ channel blockers

The loaded cells with fluo-3 AM were added three Ca^2+^ channel blockers respectively and preincubated for 10 min. These channel blockers were a non-specific Ca^2+^ channel blocker LaCl_3_, a voltage-dependent Ca^2+^ channel blocker verapamil and a putative mitochondrial and endoplasmic reticulum Ca^2+^ channnel inhibitor ruthenium red, whose final concentration were 0.2 mmol/L, 10 µmol/L and 10 µmol/L.

### Hypoosmotic or hyperosmotic stress shock and fuorescence imaging

*D. salina* cells preincubated with Ca^2+^ channel blockers were harvested by centrifugation at 5,000 g for 15 min at room temperature and then treated with hypoosmotic or hyperosmotic stress in isovolumetric fresh medium, which contained 0.5 or 4.5 M NaCl.

After treatment with hypoosmotic or hyperosmotic stress, fluorescence from *D. salina* cells loaded with fluo-3 AM was detected immediately under CLSM (Leica TCS SP5, Leica Microsystems CMS GmbH, Mannheim, Germany). *D. salina* culture was dropped on the slide with a groove that was filled with culture. The intact *D. salina* cells in the groove of the slide covered with coverslip were chosen by microscope for detection. Imaging of the cells were obtained with excitation by argon laser and monitored with an intensified CCD camera. The excitation wavelength and emission wavelength were 488 nm and 525 nm. Data of image and fluorescence were detected every 10 s under CLSM with soft LAS AF 2.1.1 build 4443 and the total time was 10 min. An intact *D. salina* cell was chosen to calculate single cell cytosolic free calcium concentration. Concentration of cytosolic free calcium was calculated from the following equation: 
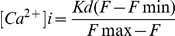
 where *Kd* is the dissociation constant of the fluo-3 AM, which is 450 nM according to the manufacturer's instructions; *F* is the fluorescence of sample; *F*min is the fluorescence in the absence of calcium and *F*max is the fluorescence of the sample at saturated calcium concentration. To obtain *F*max, the cells were exposed to a solution contained 10 µmol/L A-23187, an ionophore that is commonly used for intracellular calibration of calcium indicators. The cells were then exposed to the Ca-free solution with 1 mmol/L EGTA to obtain *F*min.

### Determination of glycerol

The algal cells in the culture containing 2.0 M NaCl at log phase were harvested by centrifugation at 5,000 g for 15 min at room temperature. The algal pellet was resuspended in fresh iso-osmotic and iso-volumetric medium, then added the three Ca^2+^ channel blockers respectively and preincubated for 10 min. Afterwards cells were harvested by centrifugation and treated with hypoosmotic or hyperosmotic shock in isovolumetric fresh medium, which contained 0.5 or 4.5 M NaCl. After 2 h, resuspended algae cells were harvested again by centrifugation for the measurement of glycerol content. According to the method of Chen et al. [21], the glycerol content in each sample was measured.

### Enzyme extraction

The algal cells in the culture containing 2.0 M NaCl were also preincubated with the three Ca^2+^ channel blockers respectively for 10 min and then treated with hypoosmotic or hyperosmotic stress for 2 h according to the method above for the extraction of enzyme and the measurement of (NAD^+^)-dependent G3pdh activity. The crude enzyme extract from cells of each sample were obtained according to the method of Chen et al. [22].

### (NAD^+^)-dependent G3pdh activity

The activity of G3pdh, catalyzing a reversible reaction, was analyzed according to the method of Wei et al. [23] with some modifications. The forward reaction mixture of 3 mL contained pH6.9 buffer solution (33.3 mmol/L Hepes, Tricine and Mes), 0.2 mmol/L NADH, 1 mmol/L DHAP and 200 µl of enzyme extract. The backward reaction mixture of 3 mL contained 50 mmol/L glycine-NaOH buffer solution (pH 10), 250 mmol/L glycerol-3-phosphate, 4 mmol/L NAD and 200 µl of enzyme extract. The reaction mixture without enzyme extract served as control. 3 mL deionized water was used as blank. G3pdh activity was assayed at 25°C after adding coenzyme and determined by spectrophotometer at 340 nm. G3pdh activity (U) is defined as the rate of per micromoles NADH oxidation or per micromoles NAD reduction micromoles per minute. Units of specific enzyme activity (U/mg) are expressed as micromoles per minute per milligram of protein.

A relationship curve of protein concentration (mg/mL) (*y*) against OD_595_ value (*x*) was plotted and the protein concentration was calculated according to the regression equation *y* = 1.5746*x*−0.0170, *R* ^2^ = 0.9969. From the relationship curve between OD_340_ and NADH concentration regression equation, the NADH concentration was obtained by determining OD_340_: *Y* = 235.84*X*+0.0118, *R^2^* = 0.99002, where *Y* represents NADH concentration (nmol/mL) and *X* represents OD_340_ value.

### Statistical analyses

Each result shown was the mean of three replicated studies. Statistical analysis of the data was performed using the program SPSS-13, and significance was determined at a 95 or 99% confidence limit.

## Results and Discussion

### Ca^2+^ concentration in *D. salina* cell

**Figure 1:**
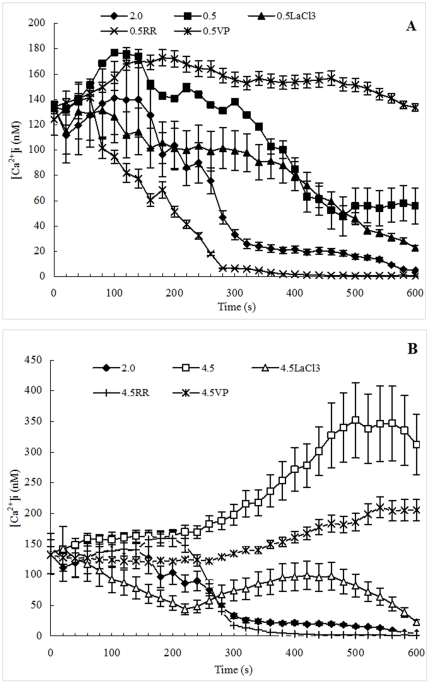
Effect of Ca^2+^ channel blockers on Ca^2+^ concentration in *D. salina* under hypoosmotic or hyperosmotic stress. A: Hypoosmotic stress; B: Hyperosmotic stress. Data points represent the means of three replicated studies in each sample, with the SD of the means (T test, p<0.05).

Figure 1 showed that the change of Ca^2+^ concentration and the effect of Ca^2+^ channel blockers on Ca^2+^ concentration in *D. salina* cell under hypoosmotic or hyperosmotic stress. Ca^2+^ concentration in *D. salina* cell under 2.0 M NaCl increased tardily and then decreased gradually after 110 s. Under hypoosmotic stress, intracellular Ca^2+^ concentration increased rapidly and then decreased gradually after 110 s, and intracellular Ca^2+^ concentration always greater than the concentration under 2.0 M NaCl (Figure 1A). After treated by LaCl_3_, verapamil or ruthenium red, it was found that the increase of Ca^2+^ concentration was less than the sample without Ca^2+^ channel blocker, indicating Ca^2+^ channel was blocked. In addition, intracellular Ca^2+^ concentration in *D. salina* cell treated by ruthenium red decreased rapidly and was less than all other samples (Figure 1A).

Under hyperosmotic stress, intracellular Ca^2+^ concentration also increased rapidly and the rising tendency was much more significant than the samples under hypoosmotic stress (Figure 1B). After treated by Ca^2+^ channel blocker, the increase of Ca^2+^ concentration was also less than the sample without Ca^2+^ channel blocker, and ruthenium red also showed the best block effect. Thus, the Ca^2+^ channels, which could be block by Ca^2+^ channel blockers used in this paper, regulated the influx.

The increase in cytosolic free Ca^2+^ concentration of *Arabidopsis thaliana* leaf cells, which was induced by exogenous application of jasmonic acid, was also inhibited by pretreatment of nifedipine, a nonpermeable L-type channel blocker [4]. In another study [24], both salinity and osmotic stress triggered transient increases in intracellular free Ca^2+^ concentration ([Ca^2+^]_i_) in cells of the nitrogen-fixing filamentous cyano-bacterium *Anabaena* sp. PCC7120. Ca^2+^ transients induced by NaCl and sucrose were completely blocked by the calcium chelator ethylene glycol-bis(β-aminoethylether)N,N,N′,N′-tetraacetic acid (EGTA) and were partially inhibited by the calcium channel blocker verapamil. However, Karimova et al. [25] found that the countertransport of Ca^2+^ and Na^+^ cross the membranes of two *Dunaliella* species (*D. salina* and *Dunaliella maritima*), the Ca^2+^ uptake depended on the intracellular Na^+^ release, and the agents blocking Ca^2+^ channels did not affect the transport of Ca^2+^ and Na^+^.

From Figure 1, it was found that the variations of Ca^2+^ concentration in the samples treated by verapamil were small and the trends were stable at the end, suggesting that verapamil may also take block effect in the process of the extrusion of Ca^2+^ from cytoplasm at later stage, which perhaps was resulted from non-specific deleterious effects of this pharmacological inhibitor.

**Figure 2:**
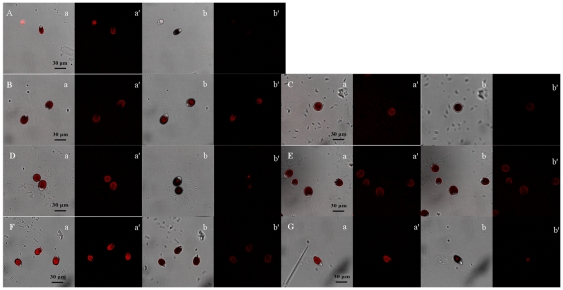
Images of *D. salina* treated by Ca^2+^ channel blockers under hypoosmotic or hyperosmotic stress. The fluorescence of Ca^2+^ in cell loaded with fluo-3 AM was excited by argon laser (excitation wavelength 488 nm, emission wavelength 525 nm), and the fluorescence of Ca^2+^ in pictures were set to be red by soft LAS AF 2.1.1 build 4443 for observation. A: 2.0 M NaCl; B: 0.5 M NaCl; C: LaCl_3_+0.5 M NaCl; D: RR+0.5 M NaCl; E: VP+0.5 M NaCl; F: 4.5 M NaCl; G: LaCl_3_+4.5 M NaCl; H: RR+4.5 M NaCl; I: VP+4.5 M NaCl. a: Image of cell shape with maximum fluorescence; a′: Fluorescence image of cell with maximum fluorescence; b: Image of cell shape with minimum fluorescence; b′: Fluorescence image of cell with minimum fluorescence. All figures are representative of three replicated studies with similar findings.

Quantitative Ca^2+^ measurements using fluo-3, fluo-4, and related indicators are hampered if there are significant Ca^2+^-independent fluorescence intensity fluctuations from cell to cell due to variations in the intracellular indicator concentration [26]. In the present study, the Ca^2+^ fluorescence was detected on single cell by using fluo-3, and all fluorescence pictures in figure 2 are representative of three replications with similar findings. Furthermore, data in figure 2 show a significant correlation (p<0.05), suggesting that the Ca^2+^-independent fluorescence fluctuations from cell to cell were minor and the conclusions were reasonable.

Figure 2 showed the changes of cell shape and fluorescence of *D. salina* cells treated by Ca^2+^ channel blockers under hypoosmotic or hyperosmotic stress. It was found that the shapes of algal cells in all samples were normal and intact, which illuminated that all blockers had no toxic or less toxic on the algal cells. It was also found that there was no marked difference on the shape of cells treated by the same salinity between the cells with blocks and the cells without blockers, indicating that these Ca^2+^ channel blockers might have no effect on the variation of cell shape.

### Glycerol content

**Figure 3:**
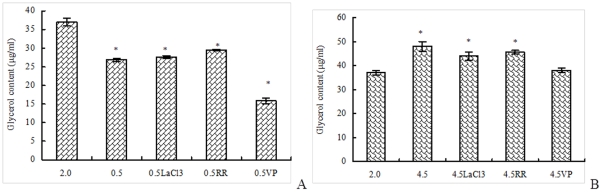
Effect of Ca^2+^ channel blockers on glycerol content in *D. salina* under hypoosmotic or hyperosmotic stress. A: Hypoosmotic stress; B: Hyperosmotic stress. Columns represent the means of three replicated studies in each sample, with the SD of the means (T test, p<0.001). The significance of the differences between the control (2.0) and test values were tested by using one-way ANOVA. *, *p*<0.05 vs control.

Under hypoosmotic stress, intracellular glycerol content decreased. After treated by LaCl_3_ or ruthenium red, the decrease of glycerol content was less than the sample without Ca^2+^ channel blocker (Figure 3A), indicating that both LaCl_3_ and ruthenium red could block the regulation of Ca^2+^-mediated osmotic signal on glycerol dissimilation in *D. salina* under hypoosmotic stress.

Under hyperosmotic stress, intracellular glycerol content increased accordingly. All the three Ca^2+^ channel blockers could block the regulation of Ca^2+^-mediated osmotic signal on glycerol synthesis in *D. salina* under hyperosmotic stress (Figure 3B) because the increase of glycerol content was always less than the sample without Ca^2+^ channel blockers.

As a result, in *D. salina* cell under salt stress, the Ca^2+^ influx may transmit some osmotic signals via Ca^2+^ channels that were related to Ca^2+^ channel blockers used in this research to regulate the glycerol dissimilation or synthesis. A similar finding had also showed that a stretch-activated Ca^2+^ channel blocker, GdCl_3_, inhibited glycerol dissimilation under hypoosmotic stress in the halotolerant alga *Dunaliella tertiolecta* [27], suggesting that the influx of Ca^2+^ from the extracellular space via the stretch-activated Ca^2+^ channels localized in the plasma membrane was required for the transduction of osmotic signal of *D*. *tertiolecta*. The rise of intracellular glycerol contents of *Dunaliella bardawil* cells under hypertonic shock was sharply decreased by low concentrations of Ca^2+^ (1 and 5 mM) but increased by high concentrations of Ca^2+^ (10 mM) [28], which also proved that Ca^2+^ could regulate intracellular glycerol content under osmotic stress.

### (NAD^+^)-dependent G3pdh activity

**Figure 4:**
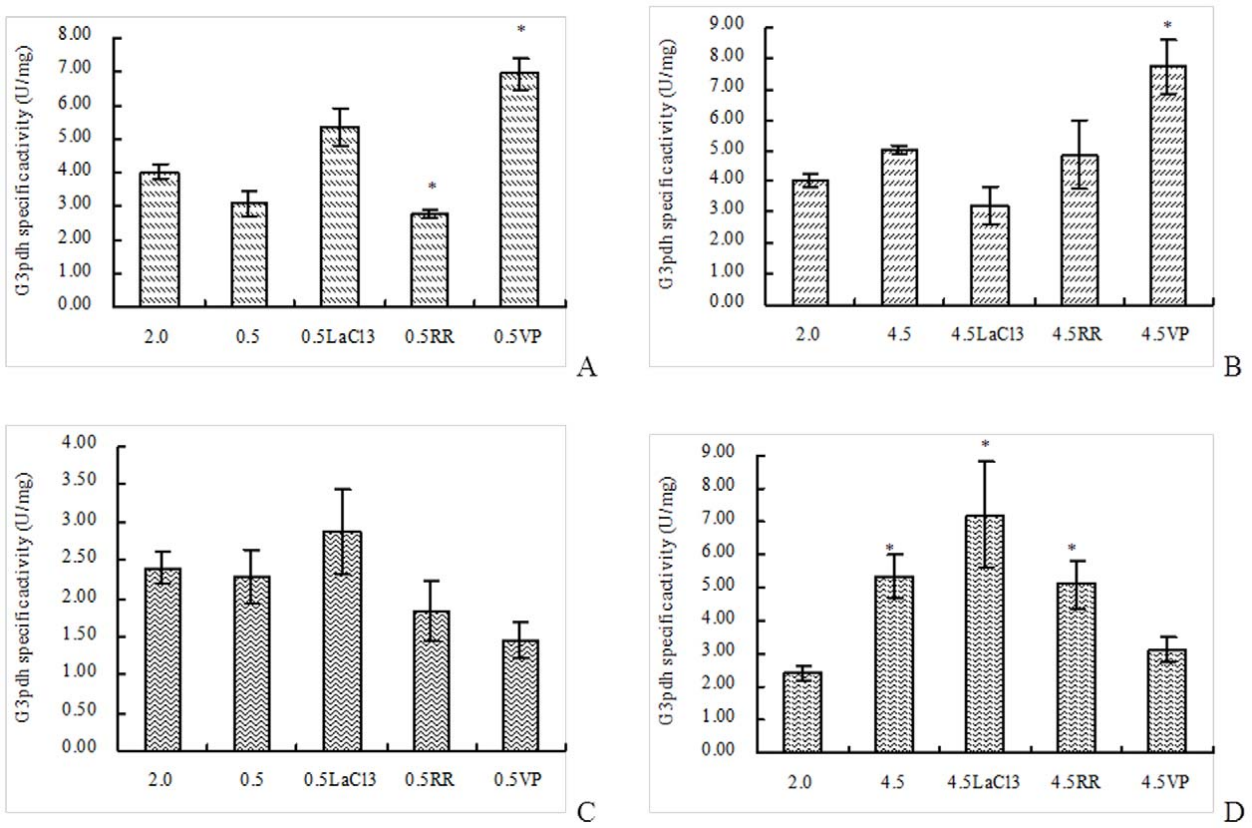
Effect of Ca^2+^ channel blockers on (NAD^+^)-dependent G3pdh activity in *D. salina* under hypoosmotic or hyperosmotic stress. A: Forward reaction activity on hypoosmotic stress; B: Forward reaction activity on hyperosmotic stress; C: Backward reaction activity on hypoosmotic stress; D: Backward reaction activity on hyperosmotic stress. Columns represent the means of three replicated studies in each sample, with the SD of the means (T test, p<0.05). The significance of the differences between the control (2.0) and test values were tested by using one-way ANOVA. *, *p*<0.05 vs control.

Under hypoosmotic stress, G3pdh forward reaction activity in *D. salina*cell reduced, but the activity in algae cell treated with LaCl_3_ or verapamil raised, which suggested that LaCl_3_ and verapamil could block the regulation of Ca^2+^-mediated osmotic signal on G3pdh forward reaction activity under hypoosmotic stress (Figure 4A). Under hyperosmotic stress, G3pdh forward reaction activity in *D. salina* cell raised. The activity in algae cell treated by LaCl_3_ reduced. The G3pdh forward reaction activity in algae cell with ruthenium red increased, but less than the sample without Ca^2+^ channel blocker (Figure 4B). Thus, it was speculated that LaCl_3_ and ruthenium red could block the regulation of Ca^2+^-mediated osmotic signal on G3pdh forward reaction activity under hyperosmotic stress.

G3pdh backward reaction activity in *D. salina* cell under hypoosmotic stress also reduced, but the activity in algae cell treated with LaCl_3_ rose. It is clear that LaCl_3_ could block the regulation of Ca^2+^-mediated osmotic signal on G3pdh backward reaction activity under hypoosmotic stress (Figure 4C). Under hyperosmotic stress, G3pdh backward reaction activity in *D. salina* cell moved up, the activity in algae cell treated by ruthenium red or verapamil also moved up but less than the sample without Ca^2+^ channel blocker (Figure 4D). Thus, ruthenium red and verapamil might played a weak role on the regulation of Ca^2+^-mediated osmotic signal on G3pdh backward reaction activity under hyperosmotic stress. Protein phosphorylation *in vitro* demonstrated that in the extract of soluble protein of *D. salina*, the activity of some protein kinases was, to some extent, dependent on the calcium concentration, and the respective mechanisms of signal transduction mediated by protein phosphorylation might not be alike [29]. Protein phosphorylation and dephosphorylation were considered as important regulatory mechanisms by which the activity of key enzymes and receptor molecules was altered within cells in response to a wide variety of external stimuli. G3pdh activity in the present study may be regulated by signal transduction mediated by some protein kinases phosphorylations, which were dependent on the calcium concentration radically.

Ca^2+^ influx via Ca^2+^ channels that could block by Ca^2+^ channel blockers used in this research might play a key role in the transduction of osmotic signal for regulating the change of (NAD^+^)-dependent G3pdh activity in *D. salina* under salt stress. Similarly, in a study by Kadota et al. [30], H_2_O_2_-induced [Ca^2+^]_cyt_ rose the expression of antioxidant enzymes. Glutathione peroxidase (GPX) and ascorbate peroxidase (APX) in tobacco BY-2 cells were inhibited by the cosuppression of NtTPC1A/B as well as Al ion, a specific blocker for NtTPC1s, the oxidative stress-responsive putative voltage-dependent Ca^2+^ permeable channels, suggesting that NtTPC1s are the major Ca^2+^-permeable channels activated by H_2_O_2_ and that Ca^2+^ influx via NtTPC1s is involved in induction of H_2_O_2_-triggered gene expression.

However, it was also found that the variations of glycerol content and G3pdh activity in samples treated by some Ca^2+^ channel blockers under salt stress were not in line with the corresponding block effect treated by other blockers (Figure 3 and Figure 4). From Figure 1, the block effect of the three blockers on the increase of Ca^2+^ concentration was ruthenium red >LaCl_3_> verapamil. Ruthenium red blocked the variations of both glycerol content and G3pdh activity except G3pdh activity under hypoosmotic stress, LaCl_3_ blocked the variations of both glycerol content and G3pdh activity except G3pdh backward reaction activity under hyperosmotic stress. Verapamil had no block effect on glycerol content and G3pdh backward reaction activity under hypoosmotic stress and G3pdh forward reaction activity under hyperosmotic stress, which suggested that the three blockers had different block effect on Ca^2+^ concentration, glycerol content and G3pdh activity. The signal transduction was mediated by Ca^2+^ influx via different Ca^2+^ channels, which were blocked by different blockers, and may have different regulatory effects on osmotic responses. Single signal transduction could not regulate whole osmotic response of glycerol and G3pdh, and there might be a synergistic effect of various osmotic signal transductions by Ca^2+^ influx via all Ca^2+^ channels or other Ca^2+^ influx mechanism. Future work should focus on the combined effects of the three blockers to determine whether they act on a single or multiple Ca^2+^ channels. By observing the combined effects, we can determine whether there is other Ca^2+^-mediated or non-Ca^2+^-mediated osmotic signal pathway in *Dunaliella* under hypoosmotic or hyperosmotic stress.

## References

[1] Plieth C (2001). Plant calcium signaling and monitoring: pros and cons and recent experimental approaches.. Protoplasma.

[2] Sanders D, Pelloux J, Brownlee C, Harper JF (2002). Calcium at the crossroads of signaling.. Plant Cell.

[3] Hetherington AH, Brownlee C (2004). The generation of Ca^2+^ signals in plants.. Annu Rev Plant Biol.

[4] Sun QP, Guo Y, Sun Y, Sun DY, Wang XJ (2006). Influx of extracellular Ca^2+^ involved in jasmonic-acid-induced elevation of [Ca^2+^]_cyt_ and *JR1* expression in *Arabidopsis thaliana*.. J Plant Res.

[5] Bothwell JHF, Ng CKY (2005). The evolution of Ca^2+^ signalling in photosynthetic eukaryotes.. New Phytol.

[6] Bothwell JHF, Brownlee C, Hetherington AM, Ng CKY, Wheeler GL (2006). Biolistic delivery of Ca^2+^ dyes into plant and algal cells.. Plant J.

[7] Zielinski RE (1998). Calmodulin and calmodulin binding proteins in plants.. Annu Rev Plant Biol.

[8] Kudla J, Xu Q, Harter K, Gruissem W, Luan S (1999). Genes for calcineurin B-like proteins in *Arabidopsis* are differentially regulated by stress signals.. Pro Natl Acad Sci USA.

[9] Sanders D, Brownlee C, Harper JF (1999). Communicating with calcium.. Plant Cell.

[10] Harmon AC, Gribskov M, Harper JF (2000). CDPKs: a kinase for every Ca^2+^ signal?. Trends Plant Sci.

[11] Kim KN, Cheong YH, Gupta R, Luan S (2000). Interaction specificity of *Arabidopsis* calcineurin B-like calcium sensors and their target kinases.. Plant Physiol.

[12] Fraser PD, Bramley PM (2004). The biosynthesis and nutritional uses of carotenoids.. Prog Lipid Res.

[13] Yan Y, Zhu YH, Jiang JG, Song DL (2005). Cloning and Sequence Analysis of the Phytoene Synthase Gene from a Unicellular Chlorophyte, *Dunaliella salina*.. J Agr Food Chem.

[14] Zhu YH, Jiang JG, Yan Y, Chen XW (2005). Isolation and Characterization of Phytoene Desaturase cDNA Involved in the β-Carotene Biosynthetic Pathway in *Dunaliella salina*.. J Agr Food Chem.

[15] Zhu Y H, Jiang JG (2008). Continuous cultivation of *Dunaliella salina* in photobioreactor for the production of β-carotene.. Eur Food Res Technol.

[16] Wang F, Jiang JG, Chen Q (2007). Progress on molecular breeding and metabolic engineering of biosynthesis pathways of C_30_, C_35_, C_40_, C_45_, C_50_ carotenoids.. Biotechnol Adv.

[17] Alkayal F, Albion RL, Tillett RL, Hathwaik LT, Lemos MS (2010). Expressed sequence tag (EST) profiling in hyper saline shocked *Dunaliella salina* reveals high expression of protein synthetic apparatus components.. Plant Sci.

[18] Avron M, Avron M, Ben-Amotz A (1992). Osmoregulation.. Dunaliella: physiology, biochemistry and biotechnology.

[19] Ben-Amotz A, Avron M (1973). The role of glycerol in osmotic regulation of the halophilic alga *Dunaliella parva*.. Plant Physiol.

[20] He QH, Qiao DR, Bai LH, Zhang QL, Yang WG (2007). Cloning and characterization of a plastidic glycerol 3-phosphate dehydrogenase cDNA from *Dunaliella salina*.. J Plant Physiol.

[21] Chen H, Lao YM, Jiang JG (2011). Effects of salinities on the gene expression of a (NAD^+^)-dependent glycerol-3-phosphate dehydrogenase in *Dunaliella salina*.. Sci Total Environ.

[22] Chen H, Jiang JG, Wu GH (2009). Effects of salinity changes on the growth of *Dunaliella salina* and the isozyme activities of glycerol-3-phosphate dehydrogenase.. J Agr Food Chem.

[23] Wei YD, Periappuram C, Datla R, Selvaraj G, Zou JT (2001). Molecular and biochemical characterizations of a plastidic glycerol-3-phosphate dehydrogenase from *Arabidopsis*.. Plant Physiol Bioch.

[24] Torrecilla I, Leganes F, Bonilla I, Fernandez-Pinas E (2001). Calcium transients in response to salinity and osmotic stress in the nitrogen-fixing cyanobacterium *Anabaena* sp. PCC7120, expressing cytosolic apoaequorin.. Plant Cell Environ.

[25] Karimova FG, Kortchouganova EE, Tarchevsky IA, Iagoucheva MR (2000). The oppositely directed Ca^2+^ and Na^+^ transmembrane transport in algal cells.. Protoplasma.

[26] Haugland RP (2002). Handbook of Fluorescent Probes and Research Products, 9th ed..

[27] Tsukahara K, Sawayama S, Yagishita T, Ogi T (1999). Effect of Ca^2+^ channel blockers on glycerol levels in *Dunaliella tertiolecta* under hypoosmotic stress.. J Biotechnol.

[28] Issa AA (1996). The role of calcium in the stress response of the halotolerant green alga *Dunaliella bardawil* Ben-Amotz et Avron.. Phyton-Annales Rei Botanicae.

[29] Chen SX, Li L, Jiao XZ (1998). Effect of osmotic shock on protein phosphorylation in *Dunaliella salina* cells.. Acta Botanica Sinica.

[30] Kadota Y, Furuichi T, Sano T, Kaya H, Gunji W (2005). Cell-cycle-dependent regulation of oxidative stress responses and Ca^2+^ permeable channels NtTPC1A/B in tobacco BY-2 cells.. Biochem Bioph Res Co.

